# External validation of the PASO prediction score for primary aldosteronism

**DOI:** 10.1530/EC-26-0255

**Published:** 2026-07-20

**Authors:** Alaa Abdelsalam, Mohammad Mahmoud Rajab Eddama, Virginia Rozalen-Garcia, Teng-Teng Chung, Umasuthan Srirangalingam, Gerard S Conway, Steven Hurel, Bernard Khoo, Stephanie E Baldeweg, Tom R Kurzawinski, Tarek E Abdel-Aziz

**Affiliations:** ^1^ Department of Endocrine Surgery, University College London Hospitals, London, UK; ^2^ Department of Head, Neck and Endocrine Surgery, Faculty of Medicine, Alexandria University, Alexandria, Egypt; ^3^ Department of Surgical Biotechnology, Division of Surgery and Interventional Science, University College London, London, UK; ^4^ Department of Endocrinology, University College London Hospitals NHS Foundation Trust, London, UK; ^5^ Endocrinology, Division of Medicine, University College London, London, UK; ^6^ Department of Targeted Intervention, Division of Surgery and Interventional Science, University College London, London, UK

**Keywords:** primary aldosteronism, adrenalectomy, hypertension, outcome prediction, PASO score, risk stratification, clinical outcomes, external validation

## Abstract

**Graphical Abstract:**

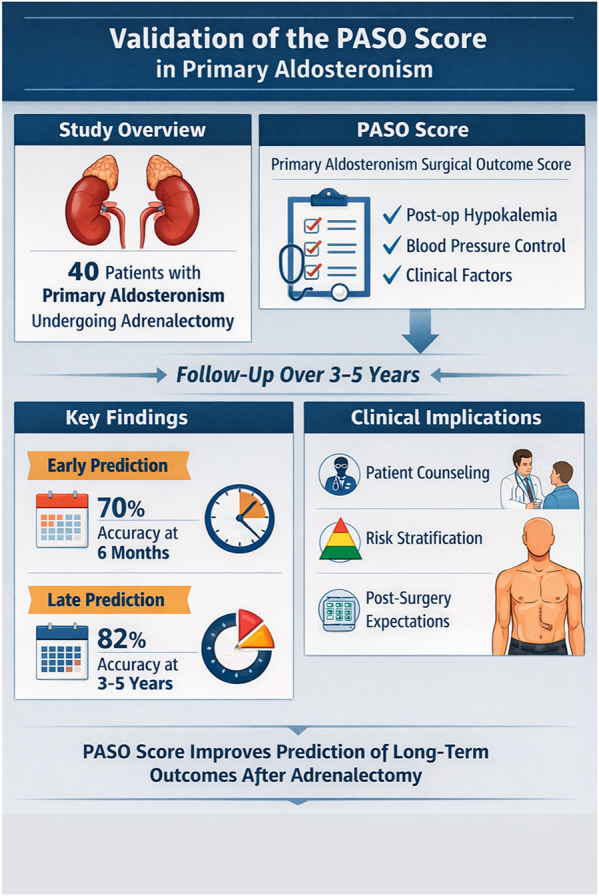

**Abstract:**

**Background**

Clinical outcomes following adrenalectomy for primary aldosteronism (PA) can vary, particularly in patients with long-standing hypertension. The primary aldosteronism surgical outcome (PASO) score was developed to estimate the likelihood of clinical success following adrenalectomy. This study aimed to externally validate the PASO score in a surgical cohort.

**Methods**

This retrospective cohort study was conducted at a single tertiary endocrine surgery centre, including patients undergoing adrenalectomy for unilateral PA over a 10-year period with a follow-up of 60 months postoperatively. The PASO score, based on six preoperative variables, was calculated for each patient. PASO score-predicted outcomes were compared with observed postoperative clinical outcomes at short-term (6 months) and long-term (60 months) follow-ups, using PASO outcome classifications as the reference standard.

**Results**

Complete biochemical cure was achieved in 39/40 patients (97.5%), while complete clinical success occurred in 9/40 (22.5%) at 6 months increasing to 14/40 (35.0%) at 60 months. Agreement between predicted and observed clinical outcomes was fair at short-term follow-up (6 months; *κ* = 0.38) and improved to substantial agreement at long-term follow-up (60 months; *κ* = 0.65), indicating stronger alignment between preoperative predictions and clinical outcomes over time.

**Conclusion**

In this patient cohort, PASO score showed modest performance for short-term outcomes but stronger agreement with long-term clinical results after adrenalectomy for PA. It may therefore be more informative for longer-term outcomes and help guide preoperative discussions with patients.

## Introduction

Primary aldosteronism (PA) is now recognised as the most common cause of secondary hypertension and is associated with higher rates of cardiovascular and cerebrovascular morbidity than essential hypertension (1, 2). Recent studies suggest that PA may be present in up to 20 per cent of patients with resistant hypertension; however, it remains underdiagnosed in routine clinical practice (3, 4).

Adrenalectomy is recommended for patients with confirmed unilateral disease and achieves biochemical cure in the majority of cases (4, 5). Accurate localisation of unilateral aldosterone excess therefore remains central to surgical decision-making. While adrenal venous sampling (AVS) is currently regarded as the reference standard, emerging CYP11B2-targeted PET imaging has shown promising accuracy and may offer a less invasive alternative. In a recent prospective study from our group, CYP11B2 PET-CT demonstrated high concordance with AVS and was associated with favourable postoperative biochemical and clinical outcomes (6). Despite high rates of biochemical cure, complete resolution of hypertension following adrenalectomy is achieved in only 25–40 per cent of patients (4, 5, 6, 7, 8).

Outcome assessment has traditionally relied on tools such as the aldosteronoma resolution score and the primary aldosteronism surgical outcome (PASO) criteria (4, 9, 10, 11). These frameworks are designed to classify clinical outcomes after surgery and are therefore not intended for preoperative risk stratification or outcome prediction.

Several preoperative scoring systems have been proposed to estimate the likelihood of hypertension resolution after adrenalectomy. However, their performance has been inconsistent across different cohorts, and no single model has demonstrated clear superiority in external validation studies (12).

Accurate assessment of surgical benefit in PA requires consideration of both short- and long-term outcomes. While early postoperative blood pressure response is frequently reported, longer-term follow-up is particularly important given the progressive and potentially reversible cardiovascular damage induced by chronic aldosterone excess, including myocardial fibrosis and vascular stiffness, which may persist despite early biochemical correction (13).

To support preoperative counselling, Burrello *et al.* developed the PASO prediction score as a tool to estimate the likelihood of hypertension resolution following unilateral adrenalectomy (14). The score is calculated using preoperative clinical variables and provides a predicted classification of clinical outcome, aligned with PASO consensus categories of complete clinical success or partial/absent clinical success (4).

The clinical benefit of adrenalectomy for PA may evolve over time, as reversal of aldosterone-mediated cardiovascular and vascular changes occurs gradually following biochemical cure. As a result, the performance of preoperative prediction tools may differ depending on whether outcomes are assessed early or at longer-term follow-up.

Although the PASO score was developed to estimate postoperative clinical success, its performance across different postoperative time points has not been well established. Given that clinical improvement may continue for several years after surgery – including sustained blood pressure reduction and decreased antihypertensive medication burden (11, 12, 13, 14, 15) – evaluation of the PASO score against both early and long-term outcomes is particularly relevant.

In this study, we aimed to externally validate the PASO score in an independent surgical cohort by comparing predicted outcomes with observed clinical outcomes at 6 and 60 months. We also assessed whether its predictive performance changes over time.

## Methods

### Study design and setting

This was a retrospective observational cohort study of patients undergoing unilateral adrenalectomy for PA at a tertiary endocrine surgery centre over a 10-year period between 2012 and 2022. The study was designed to externally validate the PASO score as a preoperative predictive tool for postoperative clinical outcomes.

Forty patients with biochemically confirmed PA who underwent surgery and had complete preoperative predictor variables and postoperative clinical outcome data were included. The study was conducted in accordance with institutional governance and audit approval frameworks.

### Patient selection

Patients undergoing unilateral adrenalectomy for PA during the study period were screened for eligibility. Diagnosis of PA was established according to the 2016 Endocrine Society Clinical Practice Guideline, based on an elevated aldosterone–renin ratio. Confirmatory testing was performed unless patients fulfilled guideline criteria for omission of confirmatory testing, namely spontaneous hypokalaemia, suppressed renin, and plasma aldosterone concentrations >20 ng/dL (550 pmol/L) (3). Disease lateralisation was determined using adrenal venous sampling, cross-sectional imaging, or a combination of both, as agreed following multidisciplinary team discussion. Adrenal vein sampling (AVS) was not routinely performed in all patients, particularly in the earlier years of the study period (2012–2022). A pragmatic approach to localisation was adopted, with CT or MRI used as the primary modality. Patients with concordant biochemical findings and a clear unilateral adrenal lesion, particularly those aged <35 years, were considered for surgery without AVS. AVS was used selectively in patients with bilateral disease, indeterminate imaging, or age >35 years.

The inclusion criteria were as follows: i) age ≥18 years; ii) diagnosis of PA made according to the 2016 Endocrine Society Clinical Practice Guideline, based on an elevated aldosterone–renin ratio followed by confirmatory testing where appropriate; iii) unilateral disease treated with adrenalectomy; iv) availability of all preoperative variables required to compute the PASO score; and v) availability of postoperative clinical outcome data sufficient to classify clinical outcomes at 6 months and/or 60 months.

The exclusion criteria were as follows: i) prior adrenal surgery; ii) missing data for one or more PASO score components; and iii) inability to determine PASO-defined clinical outcome at the relevant follow-up time point(s).

### Preoperative variables and PASO prediction score calculation

The PASO score, as described by Burrello *et al.*, is a preoperative prognostic model designed to estimate the likelihood of achieving complete clinical success following adrenalectomy. The score consists of six variables: duration of hypertension, sex, body mass index (BMI), defined daily dose (DDD) of antihypertensive medications, target organ damage (TOD – evidence of left ventricular hypertrophy on echocardiography and/or microalbuminuria), and largest adrenal nodule at imaging (Table 1). Each variable is divided into categories, which are assigned a varying number of prediction points with a maximum total of 25 points.

**Table 1:** PASO score as proposed by Burrello *et al.* (14).

Variable	Category	Points
Duration of hypertension (months)	<120	7.5
	120–239	3.5
	≥240	0
Sex	Female	3
	Male	0
Body mass index (kg/m^2^)	<24	1.5
	24–29.9	0.5
	≥30	0
Antihypertensive medication burden (DDD equivalent)	<3	6
	3–8.99	3
	≥9	0
Target organ damage*	No	3
	Yes	0
Adrenal nodule size on imaging (mm)	<13	0
	13–19	2
	≥20	4

* Target organ damage defined as the presence of left ventricular hypertrophy and/or microalbuminuria, as described by Burrello *et al.* (14).

Based on the total score, the patients are classified as more likely to achieve complete clinical success (PASO score > 16) or partial/absent clinical success (PASO score ≤ 16), reflecting predictive stratification rather than deterministic outcome prediction (14).

All variables were recorded preoperatively and used to generate a predicted outcome classification (4). For the purposes of this external validation study, the PASO score was retrospectively calculated using preoperative variables for each patient after adrenalectomy had already been performed. The score was therefore not used to guide clinical decision-making or surgical selection. Predicted PASO score classifications (complete clinical success versus partial or absent clinical success) were subsequently compared with observed postoperative outcomes (14).

### Antihypertensive medication burden

To standardise antihypertensive medication burden across different drug classes and dosages, medication use was converted to DDD equivalents using the anatomical therapeutic chemical (ATC) classification and DDD methodology of the World Health Organization Collaborating Centre for Drug Statistics Methodology (16).

The DDD represents the assumed average maintenance dose per day for a drug used for its main indication in adults and provides a standardised unit independent of formulation, strength, and prescribing patterns, allowing consistent comparison across patients.

### Outcome assessment and follow-up

Postoperative clinical outcomes were assessed using the PASO consensus criteria, which represent the international reference standard for defining clinical success following adrenalectomy for PA. These criteria classify outcomes as complete, partial, or absent clinical success based on blood pressure and antihypertensive medication requirements. In the present study, PASO criteria were used as the reference standard outcome against which the predictive performance of the preoperative PASO prediction score was evaluated (4).

Complete clinical success was defined as normalisation of blood pressure (<140/90) without the need for antihypertensive medications, whereas partial clinical success was defined as improvement in blood pressure and/or reduction in antihypertensive medication burden compared with preoperative status. Absent clinical response was defined as unchanged or worsened blood pressure and antihypertensive medication requirement. Partial and absent clinical successes were combined for analysis, consistent with the PASO score outcome framework (4). Biochemical outcomes were assessed according to the PASO consensus criteria. Complete biochemical success was defined as correction of hypokalaemia, where present, together with normalisation of the aldosterone–renin ratio.

Blood pressure measurements and antihypertensive medication use were obtained from routine postoperative outpatient clinic records and electronic patient records. In our institution, blood pressure measurements were obtained in accordance with routine clinical practice using standardised office-based measurements. Antihypertensive medication burden was quantified using DDD equivalent to enable standardised comparison across time points.

Short-term clinical outcomes were assessed at 6 months postoperatively, reflecting early postoperative blood pressure response, while long-term outcomes were assessed at 60 months following surgery, where follow-up data were available. These time points were selected to capture both early clinical response and long-term improvement associated with gradual reversal of aldosterone-mediated cardiovascular and vascular remodelling.

For each follow-up time point, observed clinical outcomes were categorised as complete clinical success or partial/absent clinical success and compared with the preoperatively derived PASO score prognostic classification.

### Statistical analysis

Statistical analyses were performed using the Statistical Package for the Social Sciences (SPSS, version 31; IBM Corp., USA) and GraphPad Prism (version 10; GraphPad Software, USA). Continuous variables were assessed for normality and compared using Student’s *t*-test, or non-parametric equivalent, as appropriate. Categorical variables were analysed using the chi-square test.

Agreement between PASO score-derived prognostic classification and observed PASO-defined clinical outcomes at short-term (6 months) and long-term (60 months) follow-ups was assessed using the Cohen’s kappa statistic. Kappa values were interpreted according to standard thresholds to describe the strength of agreement.

Discriminative performance of the PASO score was assessed using receiver operating characteristic (ROC) curve analysis, with calculation of the area under the curve (AUC) and corresponding 95% confidence interval at both follow-up time points. Sensitivity and specificity were calculated at the prespecified PASO score cut-off (>16). Positive and negative predictive values were calculated descriptively to support clinical interpretation, recognising dependence on outcome prevalence (Table 4).

Exploratory analyses using Youden’s index were performed to identify score thresholds that maximised combined sensitivity and specificity at early and long-term follow-ups; however, these analyses were considered descriptive and were not used to define the prespecified PASO score cut-off.

Model calibration was assessed descriptively by comparing observed rates of complete clinical success across PASO score prognostic strata, examining agreement between predicted likelihood of success and observed clinical outcomes.

No formal sample size calculation was performed, as this study represents an external validation of an existing prognostic score rather than model development or recalibration. The sample size was determined by the number of eligible patients with complete preoperative and follow-up data available during the study period. Analyses were therefore primarily descriptive and exploratory, with emphasis on agreement, discrimination, and calibration rather than precise estimation of predictive performance.

Statistical significance was defined as a two-sided *P*-value < 0.05 for all tests.

## Results

### Patient characteristics

Forty patients who underwent adrenalectomy for unilateral PA were included in the analysis. Baseline demographics, clinical characteristics, and biochemical response to adrenalectomy are summarised in Table 2.

**Table 2:** Patient demographics, clinical characteristics, and postoperative response.

	(*n* = 40)	Preoperative	Postoperative	*P* value
Age, years: mean (*SD*)	55 (11)			
Gender				
Male	23 (58%)			
Female	17 (42%)			
BMI, kg/m^2^: mean (*SD*)	31 (5)			
Systolic BP, mmHg: mean (*SD*)		159 (24)	129 (14)	<0.0001
Diastolic BP, mmHg: mean (*SD*)		92 (14)	79 (10)	<0.0001
Daily defined dose equivalent for antihypertensive medication: mean (*SD*)		4 (2)	2 (2)	<0.0001
Serum potassium, mEq/L: mean (*SD*)		3.3 (0.6)	4.6 (0.5)	<0.0001
ARR (aldosterone in pmol/L, plasma renin activity in ng/mL/h)		3,250 (244,1750)	152 (21, 2,700)	<0.0001
Potassium supplement dose, mEq/dL: median (range)		48 (24, 72)		
Plasma renin activity, ng/mL/h: median (range)		0.3 (0.2–0.37)		
Serum aldosterone, pmol/L: median (range)		864 (668, 1,455)		

ARR, aldosterone–renin ratio; BMI, body mass index; BP, blood pressure; SD, standard deviation.

The median duration of hypertension was 60 months (range: 6–264). Twenty-five patients (62.5%) had hypokalaemia, and target organ damage was present in 15 patients (37.5%). Adrenal nodules were identified in 36 patients, with a median size of 18 mm (range: 8–52). Adrenal venous sampling was performed in 28 patients (70%). The median operative time was 118 min (range: 57–195).

### Clinical outcomes and comparison with PASO criteria

**Figure 1:**
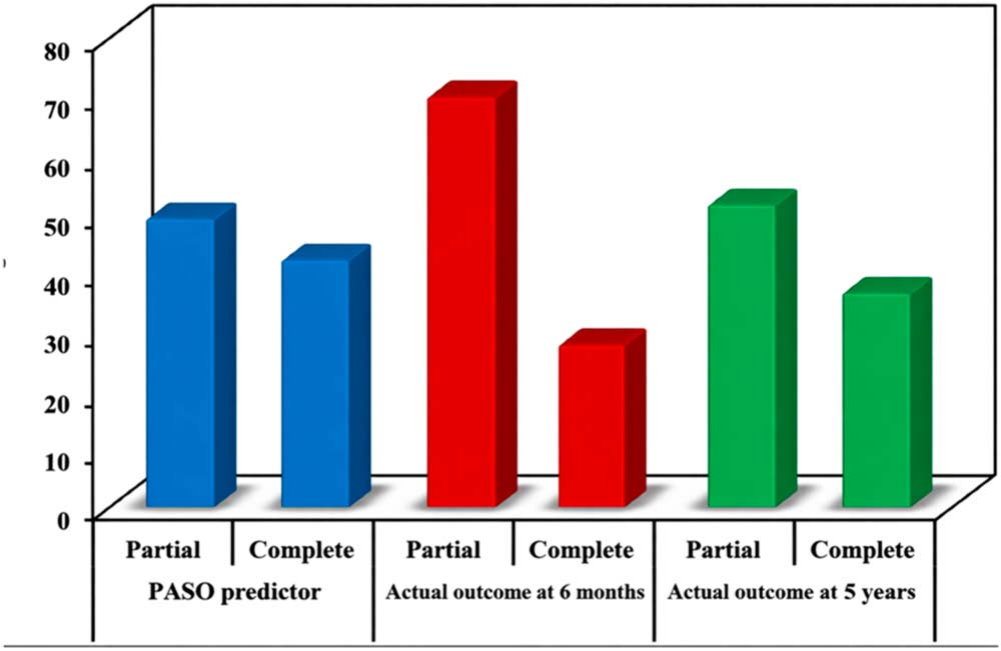
Distribution of the studied cases according to PASO score and actual clinical outcome (*n* = 40).

Using the predefined PASO score cut-off (>16), 19 of 40 patients (47.5%) were classified as likely to achieve complete clinical success, whereas 21 patients (52.5%) were classified as likely to achieve partial or absent clinical success. The PASO score demonstrated agreement with both short-term (6 months) and long-term (60 months) PASO-defined clinical outcomes, with higher concordance observed for long-term outcomes. Agreement statistics are summarised in Table 3, including Cohen’s *κ* with 95% CI and corresponding *P* values, while Fig. 1 illustrates the relationship between PASO score prognostic classification and observed clinical outcomes at both time points.

**Table 3:** PASO score agreement statistics comparison of pre- and post-adrenalectomy.

PASO score follow-up period (*n* = 40)	Observed agreement	*κ* (95% CI)	*P* value	Interpretation
Early clinical outcome (6 months)	70% (28/40)	0.38 (0.13, 0.63)	0.005	Fair/moderate
Late clinical outcome (60 months)	83% (33/40)	0.65 (0.41, 0.88)	<0.0001	Substantial

*κ*, Cohen’s kappa; CI, confidence interval; PASO, primary aldosteronism surgical outcome.

The classification performance of the PASO score at both follow-up time points is shown in Table 4. At 6 months, the score correctly classified the majority of patients, including 8 of 9 with complete clinical success and 20 of 31 without complete success. At 60 months, classification accuracy improved further, with 13 of 14 complete clinical successes and 20 of 26 partial or absent clinical successes correctly predicted.

**Table 4:** Confusion matrices comparing PASO prediction score with observed PASO clinical outcomes at 6 and 60 months.

PASO prediction score	6-month complete	6-month partial/absent	60-month complete	60-month partial/absent
Predicted complete success (>16)	8	11	13	6
Predicted partial/absent (≤16)	1	20	1	20
Total	9	31	14	26

PASO clinical outcomes defined according to PASO consensus criteria. The PASO prediction score cut-off of >16 was used to define predicted complete clinical success.

### Predictive performance of PASO score

**Figure 2:**
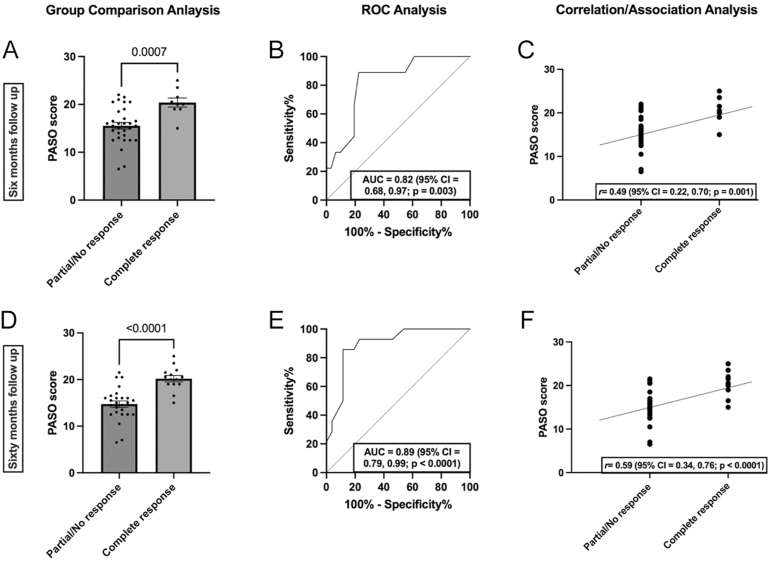
Group comparison, ROC, and association analyses demonstrating predictive performance of the PASO score at 6- and 60-month follow-ups. (A and D) Group comparison analysis demonstrating significantly higher PASO scores in patients achieving complete clinical success compared with those with partial or absent clinical success at 6 and 60 months, respectively. (B and E) Receiver operating characteristic (ROC) curve demonstrating the discriminative performance of PASO score at 6 and 60 months, respectively. (C and F) Association analyses demonstrating the relationship between PASO score magnitude and observed clinical outcome at 6 and 60 months, respectively. PASO, primary aldosteronism surgical outcome; AUC, area under the receiver operating characteristic curve; CI, confidence interval; ROC, receiver operating characteristic.

Using the historically defined PASO cut-off value of >16, the PASO score demonstrated good discriminative ability for predicting clinical response at both follow-up time points. As shown in Fig. 2, PASO scores were significantly higher in patients achieving complete clinical response compared with those with partial or no response at both 6 and 60 months.

At 6 months, ROC analysis demonstrated good predictive performance of the PASO score (Fig. 2A, B, C), with high sensitivity and excellent negative predictive value. The corresponding AUC, sensitivity, specificity, and predictive values are summarised in Table 5. These findings support the utility of PASO score in identifying patients unlikely to achieve complete response in the early postoperative period.

**Table 5:** Discriminative performance of PASO score (cut-off point of >16).

Follow-up	AUC (95%CI, *P* value)	Sensitivity (95%CI)	Specificity (95%CI)	PPV	NPV
Six months	0.82 (0.68, 0.97; 0.003)	89% (57%, 99%)	65% (47%, 79%)	42%	95%
Sixty months	0.89 (0.79, 0.99; <0.0001)	93% (69%, 100%)	77% (58%, 89%)	68%	95%

AUC, area under the curve; CI, confidence interval; PASO, primary aldosteronism surgical outcome; PPV, positive predictive value; NPV, negative predictive value.

At 60 months, the discriminative performance of the PASO score improved further, with more pronounced separation between response groups and stronger correlation with observed clinical outcomes (Fig. 2D, E, F). ROC analysis demonstrated excellent long-term prognostic accuracy, with higher sensitivity and specificity compared with 6-month follow-up, as detailed in Table 5.

Overall, Fig. 2 and Table 5 demonstrate that the PASO score, applied using the established cut-off, provides robust short- and long-term prognostic information, with greater concordance between predicted and observed outcomes at extended follow-up.

Youden’s index was calculated for completeness and not to modify the original, historically defined PASO cut-off value (>16). In this cohort, a PASO score threshold of >18.75 yielded the highest Youden’s index at both follow-up time points, with values of 0.66 at 6 months and 0.75 at 60 months.

## Discussion

In this external validation study, the PASO score performed well in predicting clinical outcomes after adrenalectomy for unilateral PA, with better performance seen at longer follow-up. While agreement with outcomes at 6 months was modest, the score showed stronger discrimination at 60 months, with an AUC of 0.89. Few external validation studies have assessed the PASO prediction score with structured long-term follow-up, and our findings suggest that it may be more useful for predicting sustained clinical benefit rather than early postoperative blood pressure response alone. To our knowledge, this represents one of the few external validation studies assessing PASO prediction score performance at 60 months.

Complete clinical resolution of hypertension after adrenalectomy has been reported in approximately 25–40% of patients in many surgical series (4, 5, 6, 7, 8), although meta-analyses have reported pooled cure rates approaching 50% depending on patient selection and follow-up duration (17). This reflects the frequent coexistence of essential hypertension and the cumulative vascular and myocardial effects of prolonged aldosterone excess, which may persist despite biochemical correction (18, 19, 20, 21). Our findings are consistent with this literature, demonstrating that although biochemical cure was achieved in almost all patients, complete clinical success occurred in a smaller proportion, particularly at early follow-up.

The PASO score was developed to support preoperative counselling by estimating the likelihood of achieving complete clinical success after adrenalectomy. While outcome assessment after adrenalectomy has traditionally relied on postoperative classification frameworks, such as the aldosteronoma resolution score and PASO criteria (4, 10), these systems are applied only after surgery and do not inform preoperative risk stratification. In contrast, the PASO score provides a preoperative estimation of the likelihood of achieving complete versus partial or absent clinical success and therefore complements postoperative PASO outcome classification. This distinction is particularly important for patient counselling and expectation management, especially in individuals with long-standing hypertension or established target organ damage.

Using PASO-defined clinical outcomes as the reference standard, preoperatively predicted outcomes generated by the PASO score were compared with observed postoperative outcomes at both short-term (6 months) and long-term (60 months) follow-ups. The PASO score classified 47.5% of patients as likely to achieve complete clinical success, whereas observed complete success occurred in 22.5% of patients at 6 months and increased to 35% at long-term follow-up.

The increase in complete clinical success observed over time paralleled the improved agreement between predicted and observed outcomes and suggests that the PASO score more closely reflects durable clinical outcomes rather than early postoperative blood pressure response alone. This temporal pattern is clinically relevant and consistent with previous studies demonstrating that blood pressure control and antihypertensive medication requirements may continue to improve for several years following adrenalectomy (11, 13). These findings emphasise the importance of incorporating long-term outcome assessment when evaluating the performance of preoperative prediction tools in PA and help explain the stronger predictive performance of the PASO score at extended follow-up.

In this context, the PASO score demonstrated utility for both early postoperative outcome prediction and estimation of longer-term clinical success. Its ability to discriminate early postoperative clinical response supports its predictive role with respect to surgical outcome, while the stronger agreement and improved discrimination observed at long-term follow-up support its prognostic value for durable clinical outcomes following adrenalectomy. Youden’s index was calculated to explore optimal discriminative thresholds within this cohort and was >18.75; however, the historically defined PASO cut-off of >16 was retained to preserve the external validity and clinical interpretability of the score.

From a clinical perspective, external validation of the PASO score supports its role as an adjunct to preoperative multidisciplinary assessment in patients with unilateral PA. By providing an estimate of the likelihood of complete versus partial or absent clinical success, the score may assist clinicians in counselling patients regarding realistic postoperative expectations, particularly in those with long-standing hypertension or established target organ damage.

A previous multicentre study examining a simplified PASO score, calculated without incorporating target organ damage, demonstrated moderate predictive performance (AUC 0.730) in an international cohort, with complete clinical success achieved in 29.5% of patients. This is particularly relevant given that some PASO score components, such as target organ damage, may not be readily available in routine clinical practice (22). Although omission of these variables resulted in a modest reduction in predictive accuracy, the simplified model remained clinically useful. This supports the role of the PASO score as a pragmatic counselling tool rather than for definitive determinant of surgical decision-making.

In the original derivation and validation study by Burrello *et al.*, the PASO prediction score demonstrated good discriminative performance, with an area under the curve of approximately 0.80 for prediction of complete clinical success. Our findings are consistent with these results and demonstrate similar or slightly improved discriminative performance, particularly at long-term follow-up where the AUC reached 0.89. This supports the external validity and generalisability of the PASO prediction score across independent surgical cohorts (14).

A recent systematic review and meta-analysis evaluating prognostic models for hypertension resolution after adrenalectomy identified the PASO score, the Utsumi nomogram, and the aldosteronoma resolution score as the most widely studied models, with broadly comparable discriminative performance across external validation studies (AUC approximately 0.77–0.81) (23).

In addition, newer models have been proposed to further refine preoperative prediction. For example, the SPAIN-ALDO multicentre registry developed a predictive model incorporating clinical variables, such as female sex, lower antihypertensive medication burden, absence of diabetes, and non-obesity, achieving an AUC of approximately 0.84 for predicting hypertension cure after adrenalectomy (24).

This study has limitations. Its retrospective design introduces potential selection and information bias, and the sample size is modest. Although long-term follow-up data were available, larger multicentre studies with prospective design would further strengthen the evidence base. In addition, while the PASO score incorporates clinically relevant preoperative variables, it does not account for postoperative factors that may also influence long-term blood pressure outcomes. Another limitation of this study is that AVS was not performed in all patients, particularly in the earlier years of the study period. As such, our cohort is not directly comparable to the original PASO study, where AVS was more consistently utilised. Furthermore, reliance on cross-sectional imaging for lateralisation may have excluded patients with non-visible aldosterone-producing adenomas. A systematic review of 950 patients demonstrated that imaging and AVS were discordant, with approximately one in five patients classified as having bilateral disease on CT or MRI subsequently shown to have unilateral, surgically curable disease on AVS (25). Our imaging-based selection strategy may therefore not have captured such cases, although the high biochemical cure rate (97.5%) in operated patients suggests that imaging-guided selection performed acceptably in those with clear unilateral lesions.

Despite these limitations, this study provides important external validation of the PASO score in an independent cohort with both short- and long-term follow-ups. The findings support its use as a practical preoperative tool to estimate the likelihood of clinical success after adrenalectomy for PA and reinforce the importance of long-term outcome assessment when evaluating surgical success.

## Conclusion

External validation in an independent cohort supports the PASO score as a useful preoperative tool for estimating clinical outcomes after adrenalectomy for PA. The score demonstrated fair agreement with short-term outcomes and substantially improved concordance with long-term PASO-defined clinical outcomes, supporting its combined predictive value for early surgical response and prognostic value for durable clinical success. These findings support the role of the PASO score in preoperative patient counselling, expectation management, and shared decision-making prior to surgery and reinforce its value as a clinically applicable predictive tool.

## Declaration of interest

The authors declare that there is no conflict of interest that could be perceived as prejudicing the impartiality of the work reported.

## Funding

This research did not receive any specific grant from funding agencies in the public, commercial, or not-for-profit sectors.

## Ethics and consent

Data used in this study were derived from a previously registered clinical audit evaluating short- and long-term outcomes following adrenalectomy for primary aldosteronism. The study was assessed using the UK Health Research Authority (HRA) decision tool and was classified as not research, and therefore, formal NHS Research Ethics Committee approval was not required. Analysis of anonymised data was conducted in accordance with local institutional audit and governance procedures.
